# The Adrenal Cortisol Response to Increasing Ambient Temperature in Polar Bears (*Ursus maritimus*)

**DOI:** 10.3390/ani12060672

**Published:** 2022-03-08

**Authors:** Emily M. Leishman, Maria Franke, Jill Marvin, Dylan McCart, Carol Bradford, Zoltan S. Gyimesi, Anne Nichols, Marie-Pierre Lessard, David Page, C-Jae Breiter, Laura H. Graham

**Affiliations:** 1 Department of Animal Biosciences, University of Guelph, Guelph, ON N1G 2W1, Canada; eleishma@uoguelph.ca; 2 Toronto Zoo, Toronto, ON M1B 5K7, Canada; mfranke@torontozoo.ca; 3 Magnetic Hill Zoo and Park, Moncton, NB E1G 4V7, Canada; jill.marvin@moncton.ca; 4 Churchill Northern Studies Centre, Churchill, MB R0B 0E0, Canada; dylan.mccart@churchillscience.ca; 5 Albuquerque BioPark, Albuquerque, NM 87102, USA; cbradford@cabq.gov; 6 Louisville Zoo, Louisville, KY 40213, USA; zoli.gyimesi@louisvilleky.gov; 7 Brookfield Zoo, Brookfield, IL 60513, USA; anne.nichols@czs.org; 8 Society of Outdoor Establishments of Quebec (Sépaq), Quebec City, QC G1W 4S3, Canada; lessard.mariepierre@sepaq.com; 9 Zoo Sauvage de Saint-Félicien, Saint-Félicien, QC G8K 0H1, Canada; david.page@zoosauvage.org; 10 Assiniboine Park Zoo, Winnipeg, MB R3R 2N7, Canada; cbreiter@assiniboinepark.ca; 11 WRG Conservation Foundation, West Montrose, ON N0B 2V0, Canada

**Keywords:** thermoregulation, climate change, glucocorticoid, HPA axis, conservation

## Abstract

**Simple Summary:**

Human-caused climate change is proceeding rapidly and providing challenges to wildlife species, especially those adapted to colder temperatures. We investigated the cortisol response of polar bears to increasing ambient temperatures to improve our knowledge of the physiology of this Arctic species with the goal of informing management in zoos and in the wild. In adult polar bears temperatures above 20 °C were associated with an increase in the hormone cortisol to accommodate increased thermoregulatory demands. This temperature threshold was surprisingly high for an Arctic-adapted species. Zoos can provide sufficient housing options to prevent overheating in polar bears exposed to warmer temperatures but our results are concerning for wild polar bears. The number of days reaching 20 °C in the Arctic has increased significantly over the past 30 years and the associated increase in thermoregulatory costs add to the physiological burden many wild polar bears are already facing with the loss of sea ice hunting opportunities. We recommend that the management of polar bears in the wild and under human care be adapted to reflect the increased cortisol concentrations associated with thermoregulatory challenges in warmer temperatures.

**Abstract:**

Our objective was to identify the upper ambient temperature threshold that triggers an increase in cortisol in response to increased thermoregulatory demands in polar bears. The results reported here include endocrine data collected over two years from 25 polar bears housed in 11 accredited zoological institutions across North America. The effects of ambient temperature, sex, age group (juvenile, adult, elderly), breeding season and humidity on fecal cortisol metabolite (FCM) concentrations (N = 8439 samples) were evaluated using linear mixed models. Ambient temperatures were placed into five different categories: <5 °C, 6–10 °C, 11–15 °C, 16–20 °C, and >20 °C. Ambient temperature and humidity had a significant (*p* < 0.05) effect on FCM concentrations with significant (*p* < 0.05) interactions of sex, age and breeding season. Once biotic factors were accounted for, there was a significant (*p* < 0.05) increase in FCM concentrations associated with ambient temperatures above 20 °C in adult polar bears. The implications of these findings for the management of both zoo and wild polar bears are discussed.

## 1. Introduction

There are two crises facing wild species and wild spaces in the 21st century. The United Nations Global Biodiversity Assessment shows alarming decline in nature with up to one million species currently facing the threat of extinction, more than at any other time in human history [1]. It lists habitat degradation, pollution and invasive species among the main drivers of nature degradation. The second crisis facing nature is human-caused climate change. The United Nations Emissions Gap Report 2021 shows that new national climate pledges combined with other mitigation measures put the world on track for a global temperature rise of 2.7 °C by the end of the century [2]. This would lead to catastrophic changes in the Earth’s climate and to potentially catastrophic impacts on wild species and their habitats. Our ability to predict, and perhaps to mitigate, to what extent these various pressures will affect the ability of individuals, species and ecosystems to survive in the long term is dependent on our understanding of their physiological processes for living in and adapting to changing environments.

The polar bear (*Ursus maritimus*) is an iconic species of the Arctic and can be considered a very visible ‘canary in the coal mine’ when it comes to the impact of climate change on the long-term survivability of a species. Last assessed in 2015, the IUCN Red List lists polar bears as vulnerable, with climate change as their main threat [3]. Polar bears rely on the presence of sea ice to hunt for food and then rely on their body’s energy reserves during the sea ice retreat in the warmer summer temperatures. Dramatic warming of the Artic in response to global climate change has resulted in the loss of sea ice and a lengthening of summer fasts, which has lowered body condition, reproduction, survival and abundance in some subpopulations; similar trends are expected throughout the Arctic as ice loss continues [4,5,6,7]. The loss of sea ice has also resulted in habitat fragmentation and loss of genetic diversity in some polar bear populations [8]. It is estimated that the current ‘business as usual’ CO_2_ emissions scenario will result in the disappearance of up to 80% of the polar bear population within 80 years [9]. 

Although there has been much discussion of how the loss of sea ice is impacting polar bears, there has been little research on how the warming environment may impact the physiology of polar bears via the thermoregulatory costs necessary to avoid hyperthermia and maintain a stable internal temperature in warmer temperatures. To accurately predict the direct physiological effects of climate change on a species, there needs to be an understanding of the thermal physiological sensitivity of the species, including how close to its thermal limits the species is living [10]. A series of early studies using polar bears trained to use a treadmill indicated that polar bears can experience exercise-induced hyperthermia even in ambient temperatures well below 0 °C [11,12]. Thus, it is possible that warming temperatures due to climate change do not just have a negative impact on the food resources and hunting ability of polar bears but may also have a negative effect on their physiology and ability to thermoregulate. Exposure to temperatures outside thermal comfort and/or tolerance ranges have been shown to have deleterious effects on animal health and reproduction in domestic and laboratory animals [13,14]. 

Our goal was to investigate thermoregulation in polar bears by utilizing polar bears housed in accredited North American zoos. This large study included long-term monitoring of physiological biomarkers as well as short-term (48 h) thermal snapshots using infrared thermography, core temperature measurements and behavioural assessments under different ambient conditions. The results presented here are focused on the adrenal endocrine responses to increasing ambient temperatures as assessed by cortisol metabolites in the feces of polar bears. Cortisol is released from the adrenal gland as part of the stress response to (perceived or real) challenges to the stability of the internal environment or homeostasis [14]. Cortisol acts on various tissues to recruit body resources to allow an individual to maintain or return to homeostasis in the face of these challenges. In mammals, thermoregulatory mechanisms are initiated to maintain internal temperature homeostasis despite changes in ambient temperature. Our objective was to identify the upper ambient temperature threshold that triggers an acute cortisol increase in response to increased thermoregulatory costs in polar bears. Zoo animals live in a relatively controlled environment that does not contain as great a diversity of stressors as those to which wild animals are exposed, which makes zoo animals ideal models to isolate how different biotic and abiotic stressors impact the physiological mediators of stress in a species. Furthermore, the non-invasive assessment of endocrine function in wildlife species was initially developed largely for zoos [15]; zoos have already contributed to our understanding of the endocrine physiology of polar bears [16].

## 2. Materials and Methods

### 2.1. Animals and Sample Collection

Fecal samples were collected from 25 polar bears housed in 11 zoological facilities across North America (Table S1) over a two-year period. All collaborating facilities were accredited by the American Association of Zoos and Aquariums and/or Canadian Accredited Zoos and Aquariums and locations of the facilities ranged from 32.7° N to 49.9° N latitude. Bears ranged from 2 to 31 years of age at the initiation of the study and included male (N_Bears_ = 13) and female (N_Bears_ = 12) bears; all bears had a body condition score ranging from 3 to 4 out of 5. Different colours of child-safe glitter or food colouring were fed to different bears housed together so fecal samples could be attributed to the correct animals; enclosures were checked daily for fecal samples (N_Samples_ = 8439). 

### 2.2. Sample Preparation

Fecal samples were collected within 24 h of defecation and were frozen at −20 °C until drying, extraction and analysis. Fecal samples were dried for three days at 65 °C in an industrial drying oven (Hotpack, Waterloo, ON, Canada), crushed into a fine powder and stored at −20 °C until extraction. Steroid hormone metabolites were extracted from an aliquot (0.18–0.23 g) of each powdered sample by agitating in 2.0 mL of 80% aqueous MeOH overnight on an orbital shaker (Junior Orbit Shaker, Lab-Line Instruments Inc., Melrose Park, IL, USA) [17]. Following agitation, the extract containing cortisol metabolites was separated from the feces via centrifugation (25 min at 2500 rpm; Beckman Model J-6M, Brea, CA, USA), stored in evaporation-proof vials (2 mL vials with an o-ring screw cap) and stored at −20 °C until immunoassay. 

### 2.3. Enzyme Immunoassay (EIA)

Fecal extracts were diluted (1:4 to 1:32) in trizma assay buffer (0.02 M Trizma, 0.300 M NaCl, 0.1% BSA; pH 7.5) prior to assay. Fecal cortisol metabolite (FCM) concentrations were quantified using a corticosterone EIA with corticosterone (3.9–500 pg/50 μL) in the standard curve. In brief, microtitre plates were coated with affinity purified goat anti-rabbit gamma globulin (25 μg/plate) dissolved in coating buffer (0.015 M Na_2_CO_3_, 0.035 M NaHCO_3_; pH 9.6) and incubated overnight at room temperature. Wells were emptied and refilled with trizma assay buffer and stored at room temperature for at least 30 min prior to use to block non-specific binding. Coated plates were washed (0.04% Tween 20) and 50 μL of diluted sample and standards were dispensed. Horse-radish peroxidase-labeled corticosterone was dispensed followed by anti-corticosterone antibody (Antibody #006; CJM Munro, UC Davis). Plates were incubated overnight at 4 °C. Plates were then washed and 200 μL of substrate solution (0.5 mL of 0.016 M tetramethylbenzidine in dimethylsulphoxide and 100 mL of 0.175 M H_2_O_2_ diluted in 24 mL of 0.01 M C_2_H_3_O_2_Na; pH 5.0) was added to each well. After incubation (45 min, room temperature) the enzyme reaction was stopped with 50 μL of stop solution in each well (3 M H_2_SO4). The optical density was measured at 450 nm (reference 595 nm). The standard curve of corticosterone ranged from 3.9–500 pg/50 μL. If sample duplicates had a percent coefficient of variation (CV) greater than 15%, samples were reanalyzed. A low and high control was assayed with each plate and the interassay CV for each was 13.38% and 6.59%, respectively. All chemicals and laboratory consumables were purchased from either Millipore Sigma Canada or Thermo Fisher Scientific Canada unless otherwise stated. Final FCM concentrations are reported in the units of ng/g dry feces.

### 2.4. Assay Validation

As an analytical validation, pools of fecal extracts from both male and female polar bears were combined and were assayed with the corticosterone EIA. To establish parallelism, serial two-fold dilutions (1:4 to 1:32) of each sample pool were tested for comparison displacement curves (Table S2). Recovery of exogenous hormone was measured by spiking a baseline diluted sample pool with corticosterone ranging from 7.8 to 250 pg (Table S3). The average percent recovery (98.3) was calculated by dividing the measured concentration of hormone by the expected concentration of hormone multiplied by 100. 

As a physiological validation, fecal samples were assayed after a known stressful event to ensure that the corticosterone assay was capable of measuring the increase in cortisol metabolites associated with an increase in adrenal activity. The known stressful event was the relocation of two of the study bears for breeding. Baseline concentrations of FCM were calculated for each bear (as described below) and a significant (2 standard deviations) increase above baseline was observed to be associated with the relocations 48 h following the relocation (Figure S1).

The baseline average FCM concentrations for each bear was calculated using an iterative process [18]. Significant stress was then defined as a value greater than two standard deviations above the baseline value. Periods where FCM levels were chronically above this level were compared to the keeper notes obtained from each facility. If a likely confounding event was occurring at the time of this elevation (i.e., bear transfer, change in social grouping, extended veterinary treatment) then the samples were excluded from further analyses. 

### 2.5. Statistical Analysis

Daily average temperature and humidity data obtained from the Weather Underground database [19] were used in the calculation. For statistical analysis, the ambient temperature was grouped into five categories: ≤5 °C, 6–9 °C, 10–14 °C, 15–19 °C, and ≥20 °C. These categories were determined based on the distribution of the data to have a similar number of samples in each category. The dates attributed to the fecal cortisol metabolites were adjusted by 2 days to reflect the estimated lag-time for the appearance of systemic changes in adrenal cortisol production in excreted feces. This lag-time was chosen based on the average time of 42.4 h that ingested iButtons™, which are used for monitoring core temperature during thermal snapshots, were retained in the study bears (Table S4). 

A generalized linear mixed model was used to analyze the effect of temperature (5 categories), humidity (%), sex (male or female), and age group (juvenile, adult, or mature) on FCM concentrations (ng/g dry feces, response variable). The interactions between temperature and sex and temperature and age group were also included in the model. Age group was determined based on the age of the bear when the study began, where juveniles were ≤5 years (N = 3 females and 4 males), adults were 6–17 years (N = 6 females and 4 males), and elderly bears were ≥18 years (N = 3 females and 5 males). These categories were determined from wild polar bear demographics [5]. Random effects included in the model were institution and bear within institution. Repeated measures were also accounted for in the model to account for multiple samples taken from the same individual throughout the study duration. A logarithmic transformation was applied to the FCM data to meet the assumption of normality. 

Due to the potentially confounding effect of breeding season on adrenal function, a sub-analysis was performed, which included only bears in the adult age group. Bears in this age group were expected to have reached sexual maturity. The model for the sub-analysis included the fixed effects of temperature (5 categories), humidity, sex (male or female), and the additional fixed effect of season (breeding vs. nonbreeding), as well as the same random and repeated effects described in the main analysis. Breeding season was defined as February to April based on previous studies in polar bears housed in zoos (see discussion), and the non-breeding season was defined as all other months of the year. 

All statistical analyses were conducted using SAS University Edition (SAS Institute, Cary, NC, USA). Significant effects were determined using α = 0.05. A covtest was conducted to determine if the included random effects (zoo and bear within zoo) were significant sources of variation. Graphical data is presented as back-transformed least-square means (LSmeans) ± SEM as per the developed model. In addition, columns illustrating the sex-specific back-transformed least-square means are included in the graphs to aid in the visualization of the interaction of sex on the relationship between ambient temperature and FCM concentrations in each age-group.

## 3. Results

Descriptive statistics for FCM concentrations for the sexes, age groups and the sex by age interaction can be found in Table 1. 

The main statistical analysis indicated a significant effect of temperature on FCM concentrations with an interaction of sex and age group on the adrenal response to temperature (Table 2). The effect of humidity on FCM concentrations was also significant in the main analysis, although the effect size was very small (log_estimate_: −0.001 ± 0.0006). The random effects of zoo (*p* = 0.0010) and bear within zoo (*p* < 0.0001) included in the analysis were significant based on the results of the covtest. 

### 3.1. Effect of Temperature on FCM Concentrations within Each Age Group

**Figure 1:**
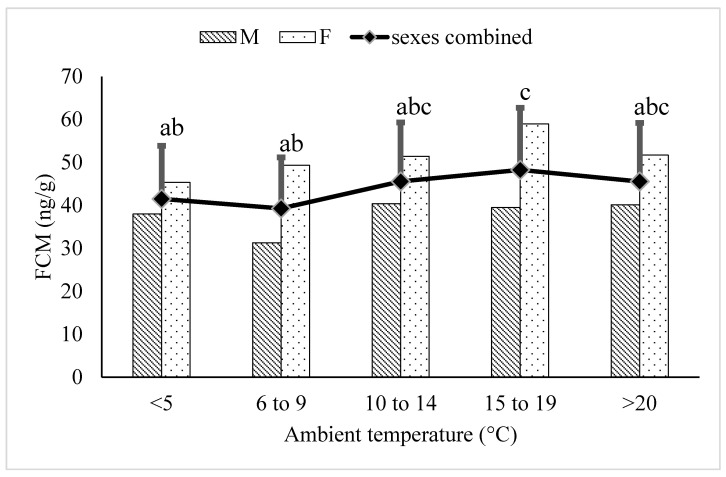
Back-transformed LSMeans ± SEM for fecal cortisol metabolite (FCM) concentrations (ng/g dry feces) within different ambient temperature categories for juvenile (≤5 years) polar bears (N_samples_ = 1613). Different subscripts represent differences between temperature categories (*p* < 0.05). Columns represent sex-specific back-transformed LSMeans for each temperature category.

In juvenile (≤5 years) polar bears, the FCM concentrations in the 15–19 °C temperature category were higher than FCM concentrations in the ≤5 °C (*p* = 0.0410) and 6–9 °C (*p* = 0.0452) temperature categories (Figure 1). The FCM levels in the ≥20 °C category were not different from the other temperature categories (*p* > 0.05). 

**Figure 2:**
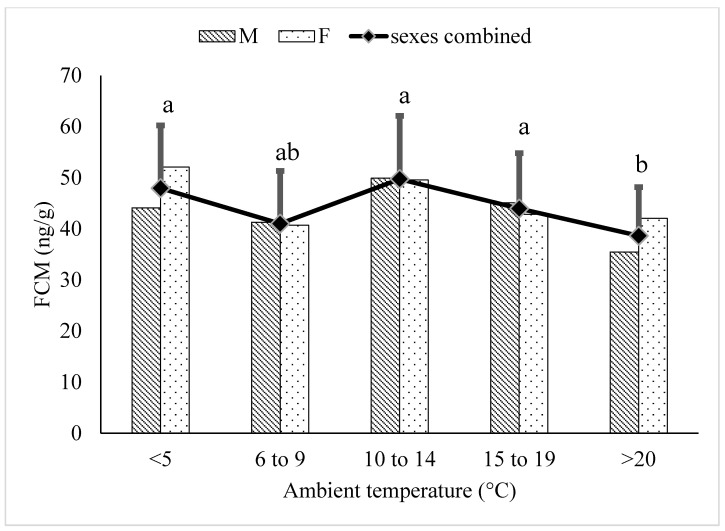
Back-transformed LSMeans ± SEM for fecal cortisol metabolite (FCM) concentrations (ng/g dry feces) within different ambient temperature categories for elderly (≥18 years) polar bears (N_samples_ = 3011). Different subscripts represent differences between temperature categories (*p* < 0.05). Columns represent sex-specific back-transformed LSMeans for each temperature category.

In elderly (≥18 years) polar bears, FCM concentrations within the ≥20 °C category were significantly lower compared to the ≥5 °C (*p* < 0.0001), 10–14 °C (*p* < 0.0001), and 15–19 °C (*p* < 0.0008) temperature categories (Figure 2). 

**Figure 3:**
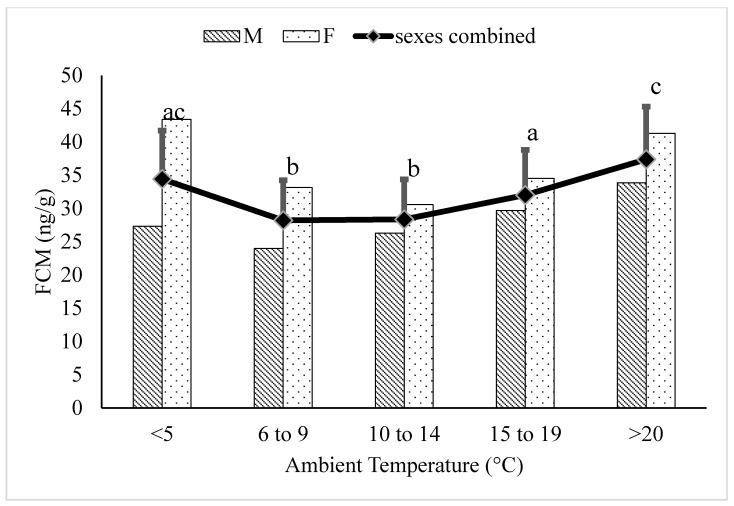
Back-transformed LSMeans ± SEM for fecal cortisol metabolite (FCM) concentrations (ng/g dry feces) within different ambient temperature categories for adult (6–17 years) polar bears (N_samples_ = 3815). Different subscripts represent differences between temperature categories (*p* < 0.05). Columns represent sex-specific back-transformed LSMeans for each temperature category.

In sexually-mature adult (6–17 years) polar bears, the FCM concentrations within the ≥20 °C temperature category were greater compared to the 6–9 °C (*p* < 0.0001), 10–14 °C (*p* < 0.0001), and 16–20 °C (*p* = 0.0003) temperature categories (Figure 3). FCM concentrations within the ≤5 °C category were not different from the ≥20 °C category (*p* > 0.05). The FCM concentrations in the 15–19 °C temperature category were greater than the 10–14 °C temperature category (*p* = 0.0354). Additionally, FCM concentrations within the <5 °C category were greater than the 6–9 °C (*p* < 0.0001) and 10–14 °C (*p* < 0.0001) temperature categories. 

### 3.2. Effect of Breeding Season on FCM Response to Ambient Temperature in Adult Polar Bears

Due to potential confounding effects of the breeding season on adrenal cortisol production, a sub-analysis was performed, including the fixed effect of breeding season (February to April) within the adult bear age category (Table 3). There was a significant interaction between temperature and season on FCM response (*p* < 0.0001). The effect of humidity on FCM concentrations was also significant in the sub-analysis, although, again, the effect size was small (log_estimate_: 0.002 ± 0.0010, *p* = 0.0188). There was a trend (*p* < 0.10) towards an effect of sex on FCM concentrations, with concentrations in adult females averaging higher than adult males.

**Figure 4:**
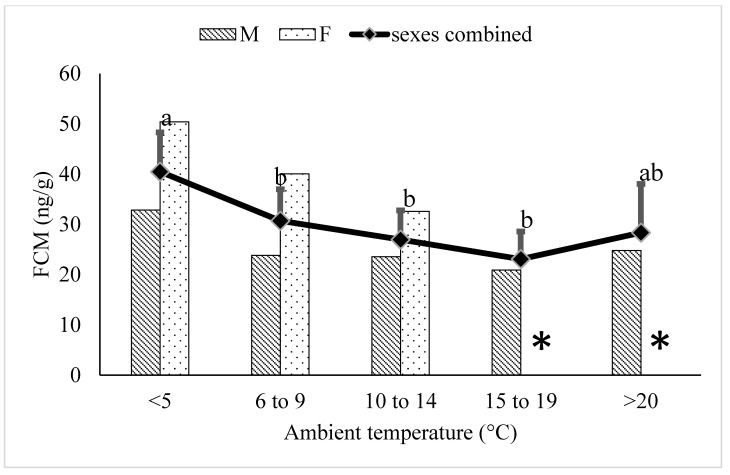
Back-transformed LSMeans ± SEM for fecal cortisol metabolite (FCM) concentrations (ng/g dry feces) within different ambient temperature categories for adult (6–17 years) polar bears during the breeding season (February to April) (N_samples_ = 1007). Different subscripts represent differences between temperature categories (*p* < 0.05). Columns represent sex-specific back-transformed LSMeans for each temperature category. Sex-specific means were not graphed for the females at higher temperature categories (*) because fewer than 3 females experienced temperatures in each of these categories during the breeding season.

During the breeding season, average FCM levels during the ≤5 °C temperature category were significantly greater than the 6–9 °C (*p* = 0.0055), 10–14 °C (*p* = 0.0011), and 16–20 °C (*p* = 0.0014) categories and were similar to concentrations associated with temperatures ≥20 °C. (Figure 4). Of the 6 adult females, 5 were housed in Canada; sex-specific means were not calculated for the columns of females at higher temperature categories because fewer than 3 females experienced temperatures in each of these categories during the February to April breeding season.

**Figure 5:**
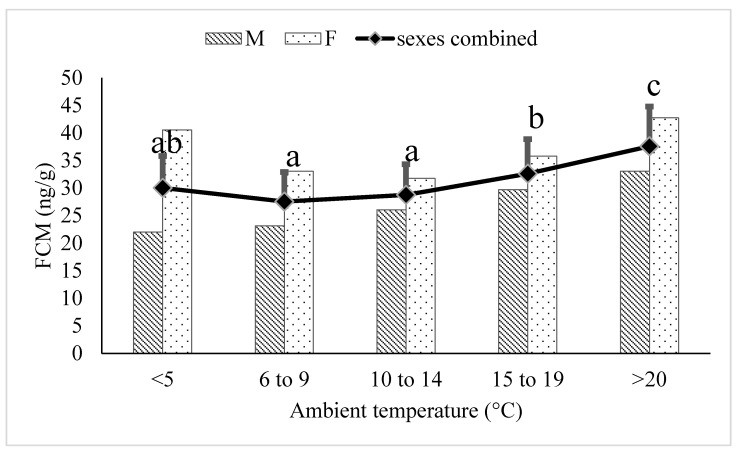
Back-transformed LSMeans ± SEM for fecal cortisol metabolite (FCM) concentrations (ng/g dry feces) within different ambient temperature categories for adult (6–17 years) polar bears during the non-breeding season (May to January) (N_samples_ = 4119). Different subscripts represent differences between temperature categories (*p* < 0.05). Columns represent sex-specific back-transformed LSMeans for each temperature category.

During the non-breeding season of May to January, average FCM levels in the ≥20 °C category were significantly greater than all other categories (*p*_≤5_ < 0.0001; *p*_6–9_ < 0.0001; *p*_10–14_ < 0.0001; *p*_15–19_ = 0.0020) (Figure 5). Average FCM levels in the 15–19 °C category were significantly greater than the 6–9 °C category (*p* = 0.0055) and the 10–14 °C category (*p* = 0.0254).

## 4. Discussion

Our objective in this study was to investigate thermoregulation in polar bears, specifically the adrenal glucocorticoid response to increasing ambient temperature. Because adrenal glucocorticoids mediate multiple physiological processes and behaviours, understanding the thermal dependencies of glucocorticoid function is important for evaluating physiological regulation and adaptation to major components of abiotic environmental variation [14]. This is particularly relevant in the age of rapid climate change; in theory, these physiological responses could be used as a predictor of population declines [20,21] and be one of a suite of assessments that could trigger specific pre-determined management plans should a quantitative threshold be reached [22]. However, the adrenal glucocorticoid response is not specific only to increased thermoregulatory costs but is a more general response to an increased stimulation of the autonomic nervous system that challenges internal stability or homeostasis. Romero et al. [23] have proposed the Reactive Scope Model to build on the traditional concepts of stress, homeostasis and allostasis. This model proposes divergent effects in four ranges of the concentrations of various physiological mediators, such as adrenal glucocorticoids, involved in the stress response. Predictive and reactive homeostasis is the range of glucocorticoid concentrations that would comprise the normal reactive scope for an individual; above these concentrations the animal would be in homeostatic overload, which is not compatible with long-term health because the elevation of glucocorticoids itself causes physiological disruption. Additionally, this model proposes an additive effect of stressors, where maintaining mediators such as glucocorticoids in the reactive homeostasis range incurs a cost; the accumulation of this cost (allostatic load) can cause wear and tear. In other words, as the animal continues to respond to a stressor, and the mediator (glucocorticoids) continues to enter the range of reactive homeostasis, the ability of that response to counteract the stressor diminishes. Thus, the Reactive Scope Model allows for the interpretation of glucocorticoid elevations with the more realistic understanding that no animal lives a stress-free life but that exposure to multiple stressors can have a cumulative effect that may negatively impact long-term health. However, it can be difficult to understand the contribution of different abiotic and biotic stressors to the allostatic load of a species in free-ranging animals because of the great diversity of potential stressors an animal is exposed to in the wild, such as predation, malnutrition, environmental challenges and inter and intra-species conflict. Animals housed in more controlled environments such as zoos can provide a clearer picture of the contributions of different abiotic and biotic stressors to the allostatic load in a species.

### 4.1. The Interactions of Sex, Age and Ambient Temperature on Adrenal Cortisol Production

We found significant effects of ambient temperature and humidity on adrenal cortisol production, as measured by fecal cortisol metabolites (FCM) (Table 2). However, we also found significant interactions of independent biotic variables with temperature on FCM concentrations; we will discuss these first. Our results demonstrated significant interactions of both sex and age with ambient temperature effects on FCM concentrations. One component of these interactions could be related to the mass of the animals. There are large mass differences between youth and adults and between the different sexes in polar bears. In the wild, male bears can reach 800 kg, while females only reach 450 kg at the time of year when they are at their maximum weight [24]. Weights were not available for all of the study bears and the weights for the 14 adult and elderly bears that we had varied considerably within a bear over the year; however, there were similar sex differences in mass. In the bears utilized in the present study, males had an average peak mass of ~490 kg, compared to ~330 kg in the females, with no overlap in the ranges of masses between the sexes; thus, the two sexes can be considered two different categories of mass. Larger mammals have less body surface area for a unit of body mass and, therefore, have slower heat loss per kilogram of body mass than do smaller mammals [25]. Thus, the large differences in mass between the sexes likely impacts their thermoregulatory responses to increasing ambient temperatures. These mass differences very likely contributed to the observed interaction of sex and age group with temperature on FCM concentrations, with the larger male polar bears displaying a sex-specific *relative* increase in FCM concentrations in response to increased thermoregulatory demands at lower ambient temperatures than the smaller females (Figure 5).

There was no increase in FCM concentrations associated with increasing ambient temperature in either the juvenile (Figure 1) or elderly (Figure 2) age groups. This may be due to a number of different factors. Both the juvenile and elderly age groups likely had physiological challenges in addition to thermoregulation that impacted adrenal cortisol production, which may have decreased the signal to noise ratio associated with increasing ambient temperature. The animals in the juvenile age group ranged from 2 to 5 years of age at the start of the study and were actively growing; those animals closer to 5 years of age may have entered physiological puberty before the end of the study, though no breeding was observed. There was also a wide range of masses in the juvenile age group, with the youngest male initially weighing ~200 kg, while one of the older males reached >400 kg at 4 years of age. 

The age of the bears in the elderly age group ranged from 18 to 31 years of age and the FCM concentrations were higher (*p* < 0.05) in elderly polar bears (47.3 ± 10.92 ng/g) than adult (31.6 ± 6.50 ng/g) or juvenile (42.9 ± 12.50 ng/g) polar bears. Most of the bears classified as elderly had exceeded the lifespan typically seen in wild polar bears by several years and suffered a variety of physical ailments associated with geriatric animals. Indeed, two of these bears were euthanized due to cancer during the study and one was euthanized shortly after the end of the sample collection period due to deteriorating age-related joint disease. It is likely that the declining health of these bears contributed to the significantly higher FCM concentrations observed in this age-group and may have decreased the signal to noise ratio of the effect of increasing ambient temperature on FCM concentrations on these bears. Alternatively, it is possible that the animals in the elderly age-group did not respond to increases in ambient temperature with a consistent increase in adrenal cortisol production to meet increasing thermoregulatory costs. Rimbach et al. [26] found that FCM levels were significantly influenced by age in monkey species, with older monkeys having higher FCM levels compared to younger monkeys. Studies investigating the effect of aging on thermoregulation suggest that an age-related decrease in cardiovascular function can impair the thermoregulatory abilities of elderly individuals due to impaired perfusion of the skin for heat exchange via convection, conduction or evaporative cooling [27]. It has also been suggested that the age-related decline in muscle mass can impact thermoregulation in elderly individuals [28]. Finally, it has been demonstrated in humans that there is a gap between thermal sensation and body temperature in the elderly even when normo-tensive [29]. Regardless, it is difficult to predict how these age-related changes in physiology would impact an adrenal response to increasing ambient temperature. The internal body temperature and surface temperature was monitored in several of our study bears during thermal snapshots as part of the larger study on thermoregulation in this species; analyses of this data may provide some insights into differences in thermoregulation across the different age groups.

### 4.2. Sub-Analyses of Sexually Mature Adult Polar Bears

Unlike juvenile and elderly animals, healthy sexually mature adults can be assumed to have a relatively stable physiology outside of changes associated with reproduction [30]. In the present study, FCM concentrations in adult polar bears during temperatures of ≥20 °C were higher than concentrations associated with lower temperatures, except for temperatures ≤ 5 °C (Figure 3). The increased FCM concentrations at temperatures below 5 °C seemed unlikely to be due to increased thermoregulatory costs given the results from early studies on thermoregulation in polar bears [11,12]. We postulated that the seasonal pattern of reproduction could contribute to increased adrenal cortisol production during the cooler temperatures. Most of the breeding observed in polar bears in zoos occurs during the cooler months of February to April [31]. Furthermore, non-invasive assessment of gonadal function in zoo polar bears shows an elevation in testosterone in males and ovarian steroids in females during this time, indicating activation of the hypothalamic-pituitary-gonadal (HPG) axis [32,33]. When we included breeding season into our model using data from bears only in the adult age-category, we found a significant interaction of breeding season on the relationship between temperature category and FCM concentrations (Table 3). Activation of the HPG axis has been shown to have a significant impact on adrenal function in a variety of mammalian species. Adrenal cortisol release increases during estrous behaviour and/or ovulation in domestic species [34,35] and humans [36] and seasonal reproductive activity has been shown to be associated with an increase in adrenal cortisol output in koalas [37] and monkeys [26]. Given that 55% of the FCM observations during the breeding season of February to April were associated with temperatures ≤5 °C, the interaction of breeding season on temperature-associated changes in FCM concentrations is not surprising (Figure 4). Applying the Reactive Scope Model, the observed impact of seasonal activation of the HPG axis on FCM concentrations should be considered within the reactive scope of the polar bears. 

In addition to the significant interaction of sex on the adrenal response to increasing ambient temperature observed in both the main and sub-analyses, there was also a trend (*p* < 0.10) towards a significant direct effect of sex on FCM concentrations in the adult polar bears, with higher concentrations in females compared to males (Table 3). One possible explanation for the trend in the difference in FCM concentrations between the sexes is that glucocorticoids are metabolized differently between the two sexes, as has been observed for corticosterone in mice and some bird species, reviewed in [38]. In other words, it is possible that the polyclonal antibody we employed had a greater cross-reactivity to the cortisol metabolites excreted by the females than different cortisol metabolites excreted by the males. However, radio-label infusion and HPLC studies have not found sex differences in the cortisol metabolites excreted in the feces of other carnivores [39]. Another possibility is that the female bears excreted a greater proportion of their cortisol metabolites in the feces versus urine when compared to males. A small, but not significant, sex difference in the proportion of cortisol metabolites excreted in the feces versus urine has been reported in domestic cats [40]. The only way to determine a sex difference in the metabolism of cortisol would be to do a radio-label infusion in polar bears of both sexes, which has not been done to date. A sex difference in fecal cortisol metabolite concentrations was not observed in a recent study on zoo polar bears using a different polyclonal antibody, but the study included only five bears in total [41]. In contrast, Oskam et al., 2004 [42] found higher serum cortisol in adult females compared to adult males in samples collected from over 200 free-ranging polar bears in Norway. These results in wild polar bears support the conclusion that the trend toward a greater concentration of fecal cortisol metabolites observed in the females compared to males in our study reflected increased adrenal activity in females rather than differences in the cortisol metabolism. While the reasons for these differences in adrenal function between the sexes is unknown, it has been observed in several mammalian species; it has been suggested that the reproductive steroids, particularly testosterone, modulate hypothalamic-pituitary-adrenal axis (HPA) activity to prevent the deleterious effects of HPA activation on reproductive function [43]. 

### 4.3. Thermoregulation in Adult Polar Bears

Our objective in this study was to identify the ambient temperature that can initiate a physiological heat stress response in polar bears as defined by an increase in adrenal glucocorticoid output. Previous studies testing adolescent polar bears on a treadmill at different ambient temperatures indicated that, as long the bears were not walking quickly there was no indication of heat stress up to 7.5 °C, the highest temperature tested [11], and high core body temperatures were rarely observed in slow-moving free-ranging polar bears in ambient temperatures between −15 °C and 15 °C [44]. However there have been no reports of the relationship between adrenal function and thermoregulatory challenges in polar bears. In our study, when the potentially confounding biotic variables of age, sex and breeding season were accounted for, there was a significant increase in FCM concentrations in response to ambient temperatures above 20 °C in adult polar bears (Figure 5). It should be emphasized that this should not be considered the upper critical temperature of the polar bear thermal neutral zone (TNZ). The theoretical TNZ of a species is the range of ambient temperatures at which body temperature regulation is achieved without regulatory changes in metabolic heat production (or changes in basal metabolic rate (BMR)) or evaporative heat loss [45]. Freely moving mammals, such as the polar bears in this study and wild polar bears, probably spend most of their lives at air temperatures outside their TNZ because the requirements specified for attainment of BMR are seldom, if ever, observed in the field [25]. Mitchell et al. [46] propose four ranges of ambient temperature where mammals are safe thermally, with the narrowest range being the TNZ. The prescriptive zone is broader than the TNZ and is where homeothermy is maintained as ambient temperature changes, despite changes in the metabolic rate. Within the prescriptive zone, metabolic rate will increase above BMR at lower ambient temperatures and evaporative cooling will be implemented at higher ambient temperatures; thus, increased energy or water intake may be required, but life is sustainable in the long term as long as these resources are available. Outside the prescriptive zone of ambient temperatures is the tolerance zone. Although some body functions may be compromised in the tolerance zone, the survival of an individual animal is not threatened, but the survival of a population may be threatened because hyperthermia can compromise reproduction. 

An increase in cortisol associated with increasing ambient temperature has been observed for a variety of taxa [17,47]. If we assume that the cross over from the prescriptive zone of ambient temperatures to the tolerance zone of ambient temperatures is the temperature at which cortisol release increases to adapt to increased thermoregulatory costs then that temperature threshold is approximately 20 °C for unrestrained polar bears in accredited zoos in North America. Initially, the 20 °C threshold may seem a surprisingly high ambient temperature for the initiation of heat stress in a species that is so well adapted to survival in the Arctic. In comparison, heat stress, including a rise in cortisol, is initiated in dairy cows at a temperature humidity index (THI) of 72 [48,49], which corresponds to a threshold temperature of only 22 °C. However, dairy cows are larger than polar bears and this extra mass may be the reason for the low ambient temperature at which heat stress is initiated in this temperate species. The THI index is a mathematical term commonly used when assessing heat stress in dairy cows that takes into account both humidity and ambient temperature, as humidity has been shown to have a significant impact on the onset of heat stress in dairy cows [48]. Our analyses indicated that ambient humidity had a significant impact on adrenal cortisol production in polar bears as well, though the effect was small. Mechanisms of evaporative cooling include panting, cutaneous water loss, including sweating, and saliva spreading or wetting of the body. Humidity can impair heat dissipation via evaporative cooling because moisture can only evaporate and dissipate heat if the water vapour pressure on the skin or in the lungs is greater than that of the surrounding air. Sweating is recognized as the most important mode of heat dissipation in cattle [50]. However, similar to many cold-adapted mammals, polar bears are covered by dense hair and heat dissipation via evaporative cooling (sweating) from the skin is likely limited. Most heavily furred mammalian carnivores use panting to facilitate evaporative heat dissipation [51]; the core temperature threshold for panting in polar bears has been reported to be 39–40 °C [52]. However, we found no interaction of humidity and ambient temperature on FCM concentrations, which precludes the development of a THI-based heat stress threshold for polar bears.

Regardless of whether or not we interpret the observed increase in FCM concentrations at 20 °C as within the normal reactive scope of polar bears as per the stress response model [23,47] or at the onset of their tolerance zone as per the thermal response model [46], there is no evidence to suggest that the polar bears in our study were exposed to thermal conditions that would send them into homeostatic overload (stress response model) or beyond their tolerance zone (thermal response model). Concentrations of FCM attributed to ambient temperatures rising above 20 °C were similar to concentrations observed with the typical temperatures (≤5 °C) associated with the breeding season (Figure 4), suggesting that they were still within the normal reactive scope of polar bears. Furthermore, additional data collected during thermal snapshots of our study bears indicated that the core body temperature did not increase above 39.5 °C (indicative of hyperthermia), even when ambient temperatures reached beyond 30 °C (unpublished observations). This suggests that the polar bears in our study remained in their thermal tolerance zone. It is possible that our zoo bears were acclimated to the warmer temperatures typically encountered in the zoo environment, as all of our bears were in captivity for at least 2 years prior to the start of the study. However, 1/3 of our study bears were orphans rescued from the wild; the rest were at most an F2 generation of a wild ancestor. This suggests that any acclimation was the result of individual experience and phenotypic plasticity and not genetic adaptation [25]. 

Repeated increases of adrenal cortisol above the predictive homeostasis range but still within the normal reactive scope of a species can still have negative consequences. “Wear and tear” is the concept that there is a cost to maintaining physiological systems in the reactive homeostasis range, so that, over time, these systems gradually lose their ability to counteract threatening and unpredictable stimuli [23]. Being subjected to thermal conditions above the prescriptive zone into the tolerance zone is also associated with negative consequences, such as a decrease in reproductive fitness [46,53]. Therefore, our results suggest that facilities that house polar bears should provide ample options for the bears to cool off at temperatures above 20 °C. All of the zoos in our study provided water features for swimming or at least submersion to their bears. Observation of behaviors during thermal snapshots of our study bears indicated that this access to water was likely critical in preventing dangerous increases in body temperature during hot days (unpublished results). The thermal conductivity of water is nearly 20 times greater than of air; thus, the convective heat rate loss in water is approximately 100–200 times greater than in air [51]. The value of submersion in water in preventing hyperthermia in polar bears was initially reported by Øritsland [54], who observed that an 80 kg polar bear cub had a stable internal temperature during extended bouts of swimming in cold sea water. This stable core temperature was maintained even during forced exercise on land for up to an hour after swimming, whereas the same exercise rapidly induced hyperthermia when not following a swim. Øritsland [54] also reported observations of hunted polar bears diving into water, even when running on land was a quicker means of escape and he speculated that this behavior was to prevent exercise-induced hyperthermia. In addition to providing cooling areas, animal managers should also schedule relocations, introductions, medical procedures and other potentially stressful events during cooler temperatures to minimize the likelihood of cortisol concentrations increasing into the homeostatic overload range. Finally, it is likely critical to provide appropriately cool denning areas for female polar bears that have been bred. Reproduction is a physiological process that has repeatedly been shown to be negatively impacted by high temperatures [53] and high cortisol concentrations associated with stressors [55]. While North American zoos are not currently breeding polar bears for re-introduction, it is quite possible that, in the face of escalating climate change, zoo populations of polar bears (and their preserved gametes) will end up being a critical resource for this threatened species.

### 4.4. Implications for Wild Bears

The goal of this study was not just to provide information on heat tolerances in polar bears so that zoos can maximize their welfare but to also provide insight into potential impacts of climate change on wild polar bears in the Arctic; our results are very concerning for wild polar bears. Given that the number of days reaching 20 °C from May to September has increased at least 30% in the past 30 years in the polar bear’s southern range (Churchill, Manitoba and Arviat, Nunavut; *weather underground*) [19], polar bears are likely experiencing greater thermoregulatory costs in addition to the increased energetic costs associated with the loss of hunting opportunities on the sea ice. The ability of polar bears to achieve energy balance is dictated by the acquisition of metabolizable energy versus expenditure from basal metabolism, thermoregulation, specific dynamic motion, reproduction, growth, and locomotion [56,57]. The zoo polar bears in our study were provided adequate nutrition to maintain body condition and were free roaming within their enclosure so they had ample opportunities to engage in thermoregulatory behaviour sufficient to prevent hyperthermia. One of the more common behaviours observed in our study bears during hot weather was minimizing activity, presumably to prevent exercise-induced hyperthermia. However, wild polar bears do not always have the luxury of minimizing their movement to prevent exercise-induced hyperthermia during warmer weather, which is a problem that is likely increasing in the face of climate change. Decreasing body condition in polar bears has been observed in the southern reaches of their range [5,6,9]; data suggest that they are unable to meet their energy demands [56]. In response to this challenge, terrestrial foraging attempts during the warmer months will likely increase as the opportunities on the sea ice decrease [58]. However, studies have indicated polar bears are not particularly energy efficient when moving at speeds necessary to chase prey on land [59], which may preclude them from effectively using land-based prey sources to sustain energy levels. Moreover, thermoregulatory costs are dramatically increased in polar bears during rapid locomotion. In studies with polar bears trained to a treadmill, bears rapidly became hyperthermic (core body temperature > 39.5 °C) whenever walking speed increased above 1.6 m/s, even when ambient temperatures were less than −25 °C [12,52]. A recent paper reported the successful hunting of reindeer by a polar bear [60] in the water, but the increasing risk of exercise-induced hyperthermia will likely prevent polar bears from effectively chasing prey on land in the warming Arctic.

There are various situations, other than foraging, that may force wild polar bears to move rapidly and to risk exercise-induced hyperthermia. Polar bears are still hunted by humans, usually using motorized vehicles, and early studies into thermoregulation in polar bears concluded that being chased by humans likely caused exercise-induced hyperthermia [11,12,52,54]. Our results support the idea that any forced rapid locomotion, such as running from human activity, could easily trigger stress hormone levels into the homeostatic overload range in addition to the exercise-induced hyperthermia in polar bears in the rapidly warming Arctic. A recent report has identified additional human recreational activities that might need to be mitigated to minimize stress hormone levels in this iconic species, particularly as opportunities for increased eco-tourism and industrial development become available [61]. 

### 4.5. Conclusions

In conclusion, our results indicate that there may be increased cortisol production in adult polar bears associated with thermoregulatory costs at ambient temperatures above 20 °C, even with reduced activity demands. Zoos need to recognize this aspect of polar bear physiology when designing housing and management protocols for polar bears in their care. In the wild, these increased thermoregulatory costs associated with warmer temperatures are likely contributing to the negative impact of climate change on polar bear physiology on top of the severe energy costs associated with the loss of sea ice hunting opportunities. Given the multitude of stressors that wild polar bears are exposed to with climate change, including thermoregulation challenges, it is quite likely that their cortisol responses to stressors are regularly increased into the homeostatic overload range, which has been suggested to decrease the likelihood of their persistence in the wild [62]. Management of wild polar bears should be adapted to reflect the ongoing changes to their habitat, including increased thermoregulatory costs. 

The reality of accelerating climate change is forcing us to rethink how we manage and interact with wildlife species. Zoos have stepped up research to maximize our understanding of polar bear biology with a new Polar Bear Master Research Plan [63] and local communities have expressed their concern for the future of wild polar bears [64]. However, the legal protection of polar bears by range nations remains inconsistent and needs to evolve to recognize the multiple and increasing stressors on this species in the wild.

## 5. Management Recommendations

Zoos should provide opportunities for bears to meet thermoregulatory needs, particularly when temperatures exceed 20 °C. Water to swim and/or submerge in is highly recommended as well as areas with cool substrates. 

Recognizing that the effect of elevated cortisol from different stressors can be cumulative, zoos should make a point of not exposing polar bears to additional stressors when ambient temperatures are above 20 °C. These potential stressors to avoid would include changes to social structure or housing and anesthesia unless critical to health of the bear. 

When attempting to breed polar bears any denning area provided to the potentially pregnant female should be kept below 20 °C. 

Unlimited drinking water should be provided at all times to ensure polar bears are hydrated sufficiently to support evaporative heat dissipation.

Policies should be enacted in range countries where necessary to outline acceptable human activities in the vicinity of polar bears to minimize disturbances that would increase their stress levels and thermoregulatory costs during the warmer months.

The classification of polar bears under species conservation laws of range countries should be upgraded to reflect the cumulative negative impact of increasing ambient temperatures on polar bear physiology.

## Figures and Tables

**Table 1:** Descriptive statistics for FCM concentrations (ng/g dry feces) for the different fixed effects and interactions included in the statistical model. Means and standard deviations (SD) presented are from the raw data before analysis.

Group	N	Mean	SD	Range
Sex				
Male	4710	59.73	74.23	1258.95
Female	3719	69.54	87.06	1580.31
Age group				
Juvenile	1611	84.84	122.44	1580.16
Adult	3812	53.00	58.10	1002.28
Elderly	3006	66.94	73.27	1055.33
Sex x Age group				
Juvenile Male	712	77.69	103.14	1255.49
Juvenile Female	899	90.50	135.59	1580.16
Adult Male	1847	48.51	58.77	1002.20
Adult Female	1965	57.23	57.16	746.39
Elderly Male	2151	63.42	73.18	1051.92
Elderly Female	855	75.80	72.79	439.90

**Table 2:** Type III Tests of Fixed Effects.

Effect	F-Value	Pr > F
Temperature Category (Temp)	3.55	0.0066
Humidity	4.42	0.0354
Sex	1.62	0.2036
Age Group	1.01	0.1823
Sex x Temp	3.70	0.0052
Age Group x Temp	13.78	<0.0001

**Table 3:** Sub-analysis in Adult Polar Bears: Type III Tests of Fixed Effects.

Effect	F-value	Pr > F
Temperature Category (Temp)	7.27	<0.0001
Humidity	5.52	0.0188
Sex	2.81	0.0937
Season	0.61	0.4348
Sex x Temp	8.04	<0.0001
Season x Temp	6.83	<0.0001

## Data Availability

Data available from the corresponding author upon reasonable request.

## Supplementary Materials

The following supporting information can be downloaded at: https://www.mdpi.com/article/10.3390/ani12060672/s1. Table S1. Identification of the latitudes of participating facilities and the study polar bears with sex and age category; Table S2. Cortisol metabolite concentrations in serially diluted fecal extract pools; Table S3. Cortisol metabolite concentrations in a fecal extract pool with added corticosterone standard; Table S4. Gut retention time (rounded to the nearest 12 hour increment) of ingested iButtons (N = 15) in polar bears (N = 11); Figure S1. Fecal cortisol concentrations in two bears (a and b) relocated for breeding purposes.
