# Supplementary material for: The Adrenal Cortisol Response to Increasing Ambient Temperature in Polar Bears (Ursus maritimus)
Source: Animals (Basel). 2022 Mar 8;12(6):672. doi: 10.3390/ani12060672 (PMC8944560; doi:10.3390/ani12060672)
Supplement: Supplementary file 1 [file animals-12-00672-s001.zip › animals-1595021-supplementary.pdf]

The adrenal cortisol response to increasing ambient temperature in polar bears (*Ursus maritimus*)

Supplemental Data

Table S1. Identification of the latitudes of participating facilities and the study polar bears with sex and age category. *Male polar bears exchanged between Facility 3 and 4 for breeding purposes.

**Facility Number**	**Latitude (°N)**	**Bear Number**	**Sex**	**Age Category**
1	49.9	1	Male	Juvenile
		2	Male	Juvenile
		3	Female	Juvenile
		4	Female	Juvenile
2	49.1	5	Male	Juvenile
3	48.7	6	Female	Adult
		7	Female	Adult
		8	*Male	Adult
		9	*Male	Adult
4	46.8	10	Female	Adult
5	45.5	11	Male	Elderly
		12	Female	Elderly
6	43.7	13	Female	Adult
		14	Female	Adult
		15	Male	Adult
7	41.9	16	Male	Elderly
		17	Male	Adult
8	39.1	18	Male	Elderly
		19	Female	Adult
9	38.3	20	Male	Juvenile
		21	Female	Juvenile
10	35.1	22	Male	Elderly
		23	Male	Elderly
11	32.7	24	Female	Elderly
		25	Female	Elderly

Table S2. Cortisol metabolite concentrations in serially diluted fecal extract pools.

Dilution	Fecal Extract Pool 1<br>Cortisol metabolites ng/ml	Fecal Extract Pool 2<br>Cortisol Metabolites ng/ml
1:4	3.236	5.200
1:8	1.700	2.527
1:16	0.830	1.356
1:32	0.424	0.698

Table S3. Cortisol metabolite concentrations in a fecal extract pool with added corticosterone standard

Corticosterone Added (ng/ml)	Cortisol metabolites ng/ml	Recovery of Added Corticosterone (%)
0	0.444	N/A
0.156	0.582	88.5
0.312	0.750	98.1
0.625	1.056	97.9
1.250	1.784	107.2
2.500	2.886	97.7
5.000	5.738	105.9
		Average = 99.2

Table S4. Gut retention time (rounded to the nearest 12 hour increment) of ingested iButtons (N = 15) in polar bears (N = 11). Enclosures were checked twice daily after iButton administration for feces. Numbers in brackets are the exact retention times for iButtons that were excreted prior to the end of the maximum temperature data recording time of 34 hours as determined by a dramatic decrease in temperature from normal body temperature to ambient temperature.

Bear Number	Administration Month	Retention Time (to the nearest 12 hour increment)
1	August	60
2	August	48
2	December	36 (32)
2	December	60
3	August	48
6	June	24 (25)
6	January	48
7	June	12 (13)
8	January	48
9	January	48
9	June	24 (21)
10	January	24 (22)
13	August	48
15	December	60
17	December	48
		Average = 42.4 hours

A

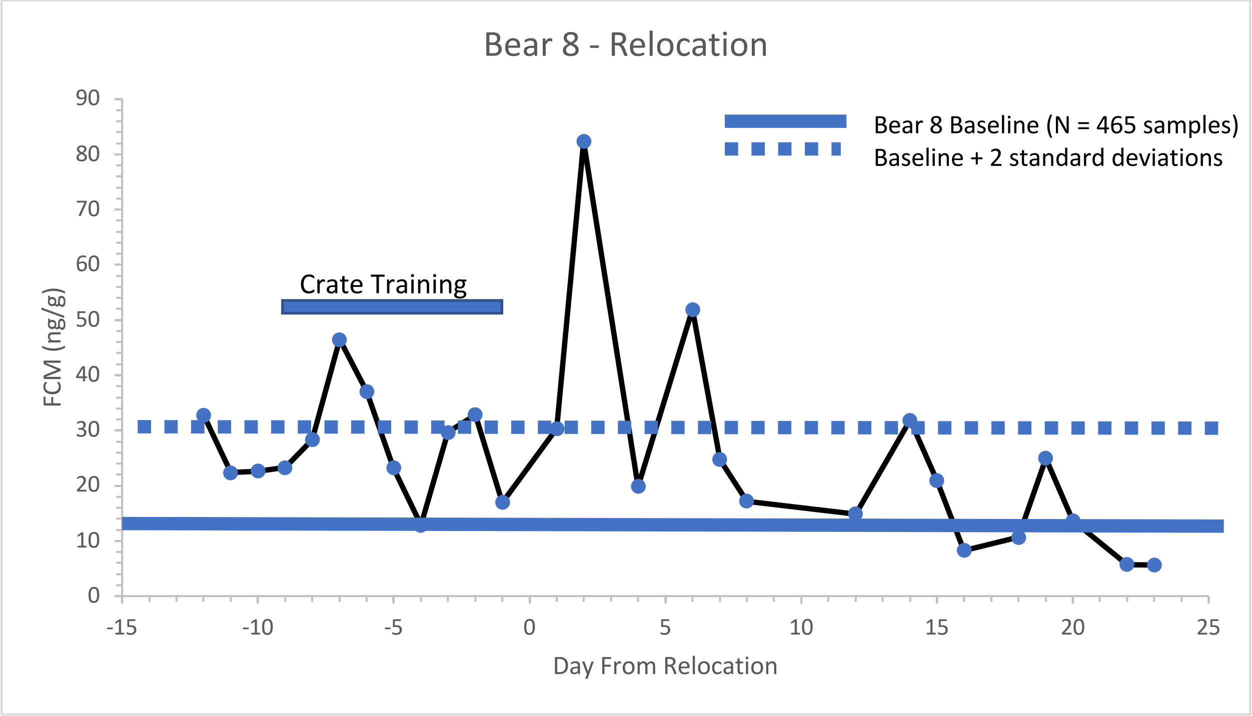

B

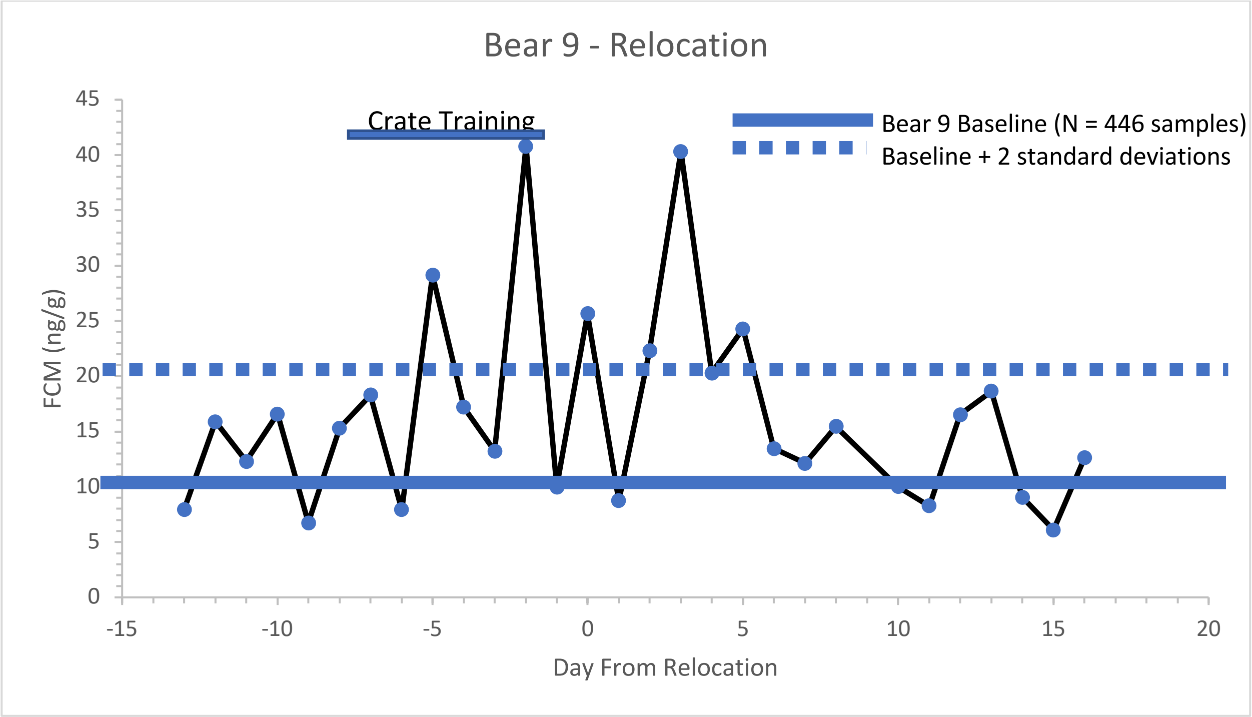

Figure S1. Fecal cortisol concentrations in two bears (a and b) relocated for breeding purposes.
